# Nonmetal‐Mediated Atomic Spalling of Large‐Area Monolayer Transition Metal Dichalcogenide

**DOI:** 10.1002/smsc.202300033

**Published:** 2023-06-30

**Authors:** Sein Kim, Seung-Il Kim, Soheil Ghods, Jin-Su Kim, Young Cheol Lee, Hyung Jun Kwun, Ji-Yun Moon, Jae-Hyun Lee

**Affiliations:** ^1^ Department of Energy Systems Research Ajou University Suwon 16499 Republic of Korea; ^2^ Department of Materials Science and Engineering Ajou University Suwon 16499 Republic of Korea

**Keywords:** atomic spalling, germanium, monolayers, transition metal dichalcogenides, wet etching

## Abstract

Transition metal dichalcogenides (TMDCs) have attracted intense interest; however, despite the considerable effort of researchers, a universal manufacturing method that can guarantee both high material quality and throughput has not been realized to date. Herein, a universal approach to producing high‐quality monolayer TMDCs on a large scale via germanium (Ge)‐mediated atomic spalling is presented. Through the modified analytic model, the study verifies that the thin Ge film could be a suitable stressor that effectively reduces the crack propagation depth at the sub‐nanometer range. In particular, an acid‐etching process is not required in the overall atomic spalling process due to the water‐soluble nature of the Ge, enabling it widely applicable to various TMDCs. Under the optimized spalling conditions, a millimeter‐sized monolayer of stable MoS_2_, as well as unstable MoTe_2_, is successfully achieved. Through detailed spectroscopic and electrical characterizations, it is confirmed that the proposed methodology for obtaining large‐area atomic layers does not introduce any significant structural defects or chemical contaminations.

## Graphical abstract

Millimeter‐sized monolayer transition metal dichalcogenides (TMDCs) are successfully separated from bulk van der Waals crystals via a Ge‐mediated atomic spalling approach. Because of the water‐soluble nature of the Ge, the acid‐based etching process that can induce physical damage to chemically unstable TMDCs is not applied in the overall atomic spalling process, allowing for high‐throughput manufacture of high‐quality monolayer TMDCs.

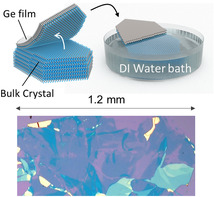

## 1 Introduction

There has recently been a substantial increase in research regarding the atomic layer of 2D materials (2DMs), which offer significant advantages over their bulk counterparts due to their exceptional electrical, optical, and mechanical properties.^[^ ^1^, ^2^, ^3^, ^4^, ^5^ ^]^ Among these materials, monolayer TMDCs have emerged as desirable elements for a wide range of applications, including electronics, photonics, sensors, and energy devices.^[^ ^6^, ^7^, ^8^, ^9^, ^10^ ^]^ Although a proof of concept of functional devices based on TMDCs has been demonstrated, there is yet to be a known scalable materials production method that can achieve both high material quality and throughput production.

Chemical vapor deposition (CVD) is considered to be a promising synthetic approach to producing wafer‐scale monolayer TMDCs with high controllability.^[^ ^11^, ^12^, ^13^ ^]^ Nevertheless, the formation of structural defects during the growth process is inevitable during CVD, thus degrading the inherent properties of TMDCs and the overall device performance.^[^ ^14^, ^15^ ^]^ Meanwhile, mechanical cleavage approaches have emerged as the simplest way to obtain the best‐quality monolayer TMDCs to date. However, the size of isolated monolayer flakes obtained using such an approach is generally limited to tens of micrometers, and the yield is very poor, which restricts its usage for academic purposes.^[^ ^16^, ^17^ ^]^

Recently, a modified exfoliation approach has been developed that combines the advantages of both CVD and the exfoliation method.^[^ ^18^, ^19^, ^20^ ^]^ Several research groups, including ours, have used this approach to successfully obtain millimeter‐sized monolayer TMDCs using a thin metal film.^[^ ^21^, ^22^, ^23^, ^24^, ^25^, ^26^, ^27^, ^28^ ^]^ For example, Huang et al. achieved monolayer TMDCs from bulk crystals by leveraging the strong affinity between Au atoms and chalcogen atoms.^[^ ^21^ ^]^ In another study, Moon et al. reported an atomic spalling method, known as the controlled crack propagation technique, by adjusting the interfacial toughness and the internal stress of the metal film.^[^ ^28^, ^29^ ^]^ However, according to the conventional process of modified exfoliation techniques, a wet‐etching process based on strong acids or iodine to remove the top or bottom metal layers (Au or Ni) is needed, which is environmentally harmful and cannot be applied to chemically unstable TMDCs (e.g., MoTe_2_).^[^ ^30^ ^]^

Here, an effective and universal TMDCs monolayer manufacturing method, on a macroscopic scale, is presented. Using Ge as a stressor film, controlled atomic spalling of monolayer TMDCs from bulk van der Waals (vdW) crystals is induced. Ge can serve as a suitable stressor material for atomic spalling because of its small interfacial toughness (γ) with vdW materials (γ_Ge‐vdW_ = 32 meV atom^−1^), which is similar to the interfacial toughness within vdW materials (γ_vdW‐vdW_ = 25 meV atom^−1^), which could effectively reduce the spalling depth of 2DMs to a few atoms thick.^[^ ^31^, ^32^ ^]^ Above all, the water‐soluble nature of Ge means it does not require a wet chemical etching process, thus making it widely applicable to various TMDCs. By utilizing a 70 nm‐thick Ge stressor, millimeter‐scale monolayer TMDCs (MoS_2_ and MoTe_2_) were successfully separated from the crystals. The spalled monolayer does not exhibit any intrinsic defects or chemical contamination, as confirmed by spectroscopic and electrical characterization. It is believed that the Ge‐mediated atomic spalling approach to achieve high‐quality and large‐scale monolayer TMDCs will pave the way for scaling up the production of 2DMs and accelerate both their fundamental research and industrial applications.

## 2 Result and Discussion

### 2.1 Analytic Model for Ge‐Mediated Atomic Spalling

**Figure 1:**
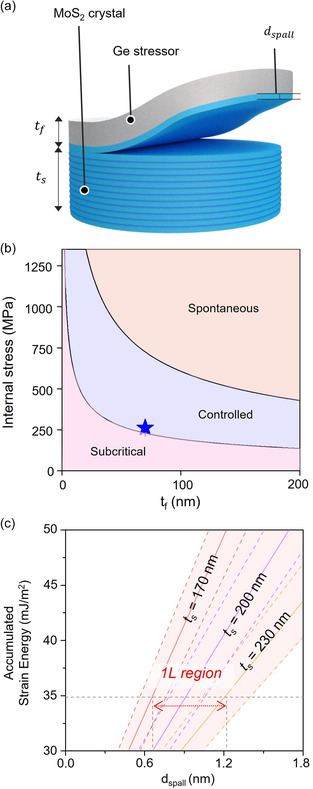
a) Schematic illustration of the controlled spalling process. b) Plot of the stressor thickness versus internal stress of the Ge stressor. The study confirmed that the internal stress of the 70 nm‐thick Ge film is in the controlled spalling window (blue star). c) Plot of the accumulated strain energy in the Ge/MoS_2_ bilayer system versus spalling depth. The spalling depth is determined by the MoS_2_ thickness in the fixed thickness of a 70 nm Ge stressor.

To investigate critical spalling conditions in the Ge/TMDCs bilayer system, theoretical studies were first conducted based on the modified Suo–Hutchinson (S–H) model.^[^ ^33^, ^34^ ^]^ The intrinsic, well‐known properties (i.e., Young's modulus and Poisson's ratio) of MoS_2_ crystal were used to determine whether and how spalling depth (*d*_spall_) is dependent on the internal stress of the Ge stressor (**Figure** **1**a and Notes S1 and S2, Supporting Information). The estimated process window for controlled spalling of the MoS_2_ crystal is depicted in Figure 1b. When the controlled spalling condition is satisfied, a crack initiates from the edge of the MoS_2_ crystals upon the application of a gentle external force to the Ge stressor. This crack then propagates downward due to the mixed‐mode fracture of *K*_I_ (vertical stress direction) and *K*_II_ (lateral stress direction).^[^ ^33^, ^34^ ^]^ Under thermodynamic equilibrium conditions, where *K*_II_ becomes zero, the total accumulated strain energy of the Ge/MoS_2_ bilayer system reaches an equilibrium with the binding energy of MoS_2_, at which point the crack no longer propagates downward but grows parallel to the interface between MoS_2_ and the Ge stressor. Consequently, the top MoS_2_ layers can be selectively separated from the crystals. The relationship between the *d*_spall_ of MoS_2_ and the internal stress (*σ*_f_) of Ge can be expressed as follows.^[^ ^28^ ^]^

(1) $$\sigma_{f}=\sqrt{\frac{\gamma }{\frac{\left({\text{1}-v}_{s}\right)}{{\text{2}Y}_{s}}\frac{t_{f}^{2}}{t_{s}}\left[12{\left(\frac{d_{\text{spall}}}{t_{s}}\right)}^{3}-\text{24}{\left(\frac{d_{\text{spall}}}{t_{s}}\right)}^{2}+\text{16}\left(\frac{d_{\text{spall}}}{t_{s}}\right)\right]+\frac{\left({\text{1}-v}_{f}\right)}{{\text{2}Y}_{f}}t_{f}}}$$

where ν_s_, *Y*_s_, *t*_s_, *t*_f_, ν_f_, and *Y*_f_ are Poisson's ratio of MoS_2_, Young's modulus of MoS_2_, the thickness of MoS_2_, the thickness of the Ge film, Poisson's ratio of Ge, and Young's modulus of Ge, respectively. In the deposition system, it was experimentally confirmed that the *σ*_f_ of the Ge film with a thickness of 70 nm is about 262 ± 10 MPa, which is within the controlled spalling region criteria (blue star symbol in Figure 1b). Therefore, when the *t*_f_ is 70 nm, it is expected that the value of *d*_spall_—which is the point at which the binding energy of MoS_2_ is balanced with the total accumulated strain energy—is within a range of monolayer MoS_2_ thickness when *t*_s_ is between 170 and 230 nm (Figure 1c).

### 2.2 Atomic Spalling of Monolayer MoS_2_

**Figure 2:**
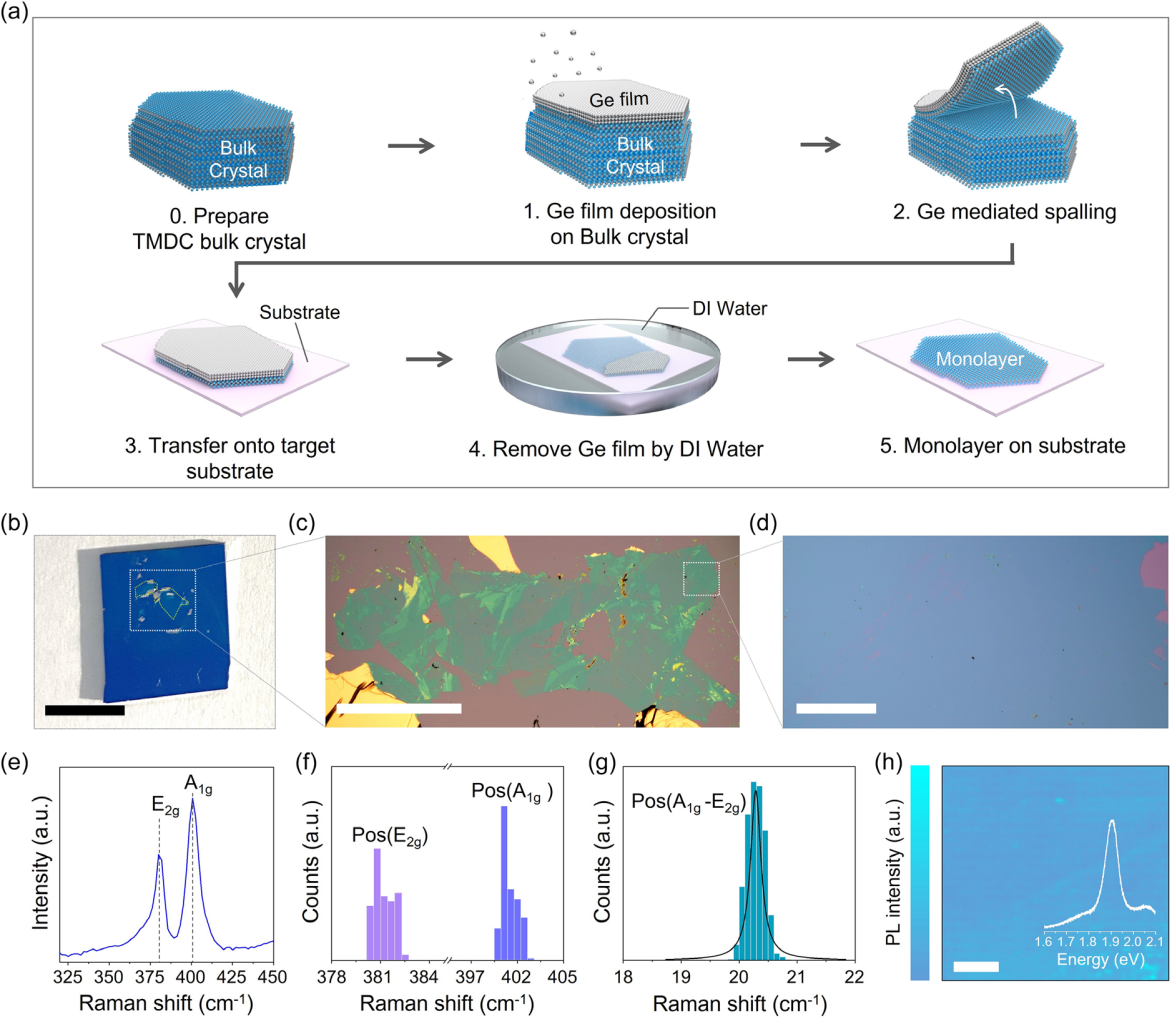
a) Schematic illustration of the Ge‐mediated atomic spalling process. b) Photograph of spalled monolayer MoS_2_ on 300 nm SiO_2_/Si substrate. The scale bar is 5 mm. c,d) Low‐ and high‐magnification OM images of large‐scale spalled monolayer MoS_2_. The scale bars are 1 mm and 100 μm, respectively. e) Representative Raman spectra of spalled monolayer MoS_2_. f,g) Histogram of the Raman peak position map for the E_2g_ peak and A_1g_ peak, and Raman peak position difference Pos (A_1g_–E_2g_) maps for monolayer MoS_2_. h) PL intensity maps at fixed positions of 1.90 eV for monolayer MoS_2_. Inset shows representative PL spectra of monolayer MoS_2_. Scale bar: 10 μm.

Based on the theoretical predictions, atomic spalling of the topmost monolayer of MoS_2_ from the thick MoS_2_ crystal was conducted. **Figure** **2**a shows the detailed process of Ge‐mediated atomic spalling. First, a cleaved MoS_2_ substrate with a thickness of 192 ± 27 nm was prepared, and a 70 nm‐thick Ge film was deposited onto a fresh MoS_2_ surface (Figure S1, Supporting Information). It was confirmed that the internal stress of the as‐deposited Ge film is in the range of 262 ± 10 MPa, which allows for the crack propagation depth within MoS_2_ monolayer thickness to be controlled. Spalled monolayer MoS_2_ is then transferred onto a target substrate (e.g., rigid SiO_2_/Si substrate and transparent flexible substrate), and the Ge film is subsequently completely washed away by dipping the sample in a clean deionized (DI) water bath (Figure 2b and S2, Supporting Information). Figure 2b shows a photograph of spalled monolayer MoS_2_ on the 300 nm SiO_2_/Si substrate. A continuous MoS_2_ monolayer with a lateral size of a few millimeters was successfully separated from the thick MoS_2_ crystal without physical or chemical damage (Figure 2c,d, and S3, Supporting Information).

The quality of the spalled monolayer MoS_2_ samples was verified through Raman and photoluminescence (PL) spectroscopy analysis. Figure 2e–g shows the representative Raman spectra and histogram for the spalled monolayer MoS_2_. The strong E_2g_ and A_1g_ peaks, which are associated with the out‐of‐plane and in‐plane vibration modes of MoS_2_, respectively, are located at the nearly fixed positions of ≈381.2 and ≈401.5 cm^−1^, respectively, with a position difference of around ≈20 cm^−1^.^[^ ^35^, ^36^ ^]^ The E_2g_ peak position in the spalled sample was found to be slightly redshifted compared to the tape‐exfoliated monolayer MoS_2_ (Figure S4, Supporting Information). This observation indicates that the spalled monolayer is under tensile strain, which is a typical feature of spalled materials.^[^ ^29^ ^]^ The intensity of the defect‐related peaks at ≈227 cm^−1^ in spalled monolayer MoS_2_ was negligible (Figure S5, Supporting Information).^[^ ^37^ ^]^ Moreover, strong PL intensity was acquired at 77 K and observed at 1.90 eV, as shown in Figure 2h and S6, Supporting Information.^[^ ^38^, ^39^ ^]^ The full width at half maximum (FWHM) of the E_2g_ peak and PL peak were 4.8 ± 0.54 cm^−1^ and 0.12 ± 0.016 eV, respectively, which were comparable values with those of the tape‐exfoliated monolayer MoS_2_ (Figure S7, Supporting Information). The above results suggest the thickness uniformity and high crystallinity of spalled MoS_2_ and support that the study's Ge‐assisted spalling approach is an effective method for obtaining high‐quality monolayer TMDCs on a large scale.^[^ ^40^, ^41^ ^]^

### 2.3 Atomic Spalling of Unstable Monolayer MoTe_2_

One of the key benefits of the study's approach is that it does not require a chemical‐based wet etching process. While a diverse range of vdW materials has been discovered, and although its large‐area monolayers have been successfully obtained through a modified exfoliation approach, chemically unstable TMDCs still pose difficulties in achieving large monolayers because unavoidable surface oxidation and physical damage occur during the metal film removal process. For example, monolayer MoTe_2_, which has a rich phase structure with important application potential, has not been realized in a large area or freestanding form using a recently modified exfoliation method. To this point, it has only been implemented on a metal substrate, and subsequent metal removal steps were needed for transfer onto the target substrate. Here, the study spalled chemically unstable MoTe_2_ via a water‐soluble Ge‐mediated spalling method. The entire spalling process only involves DI water, which prevents degradation of the intrinsic properties of MoTe_2_ throughout the procedure (Figure S8 and S9, Supporting Information).^[^ ^30^, ^42^ ^]^

**Figure 3:**
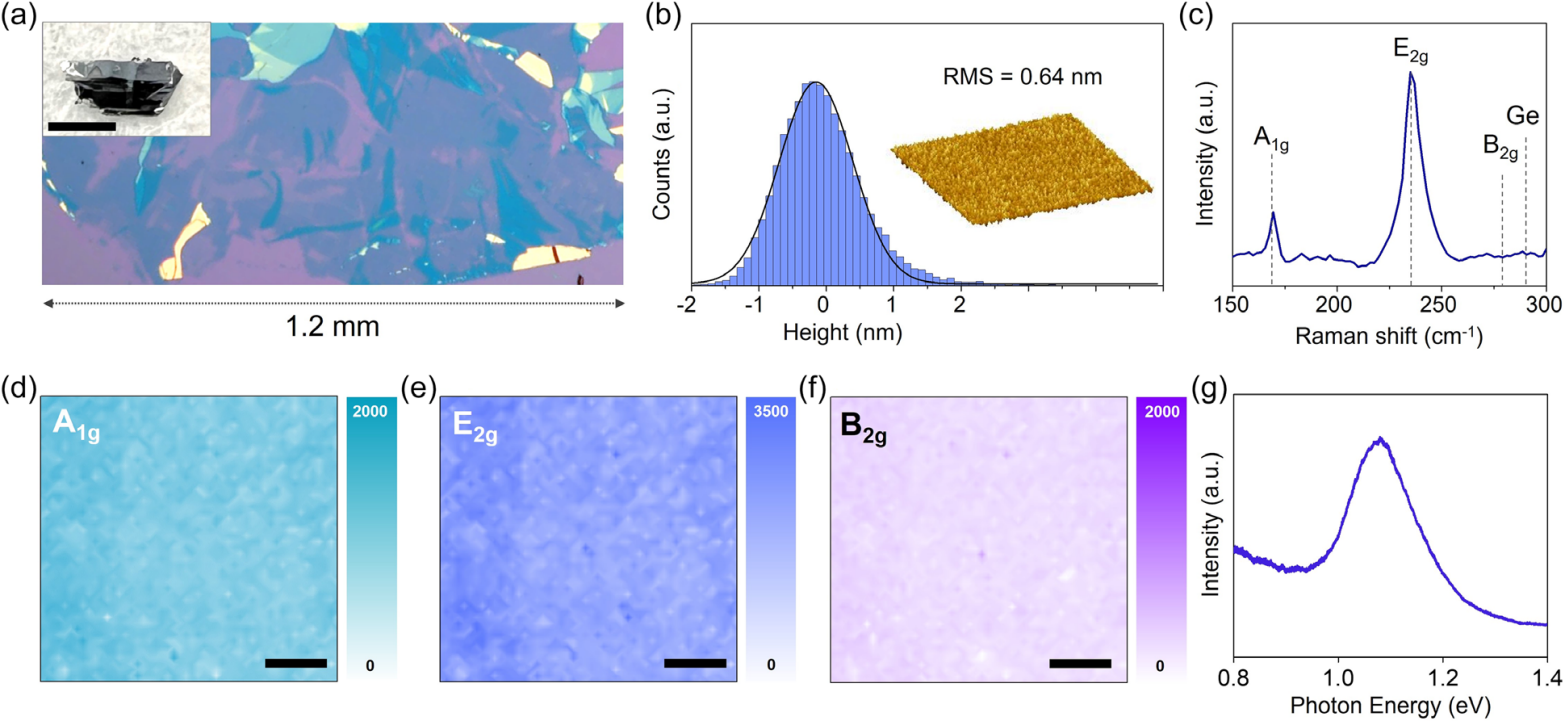
a) Optical image of large‐scale spalled monolayer MoTe_2_. The inset in (a) shows starting crystals. Scale bar: 500 μm. b) Height profile and 3D AFM image of spalled monolayer MoTe_2_ scanned over 25 μm^2^. c) Representative Raman spectra of spalled monolayer MoTe_2_. d–f) Raman intensity maps for A_1g_, E_2g_, and B_2g_ peaks of monolayer spalled MoTe_2_. The scale bar is 10 μm. g) PL spectrum of monolayer spalled MoTe_2_ under 532 nm excitation.

Since the mechanical properties of MoTe_2_ are similar to those of MoS_2_, depositing a 70 nm Ge film enabled the peeling off of the topmost MoTe_2_ monolayer from the bulk crystal, thus yielding a monolayer of MoTe_2_ with a lateral dimension of 1.2 mm, which is determined by the starting crystal (**Figure** **3**a).^[^ ^43^, ^44^ ^]^ Microscopy and spectroscopy analyses confirmed the cleanliness and lack of surface damage to the monolayer MoTe_2_ after the DI water dipping process. Atomic force microscopy (AFM) images of the spalled MoTe_2_ on a 300 nm SiO_2_/Si substrate showed a uniform and smooth surface without any polymeric residue or physical defects such as cracks, tearing, and folding (Figure 3b).^[^ ^45^ ^]^ The root mean square roughness further supported that the flatness of MoTe_2_ is similar to that of exfoliated 2D materials on a SiO_2_/Si substrate.^[^ ^46^ ^]^

The study next conducted Raman and PL spectroscopy measurements for spalled MoTe_2_. A strong E_2g_ mode (236 cm^−1^) and a clear single peak in the A_1g_ mode (169 cm^−1^) were observed, without Davydov splitting generated by interlayer interactions (Figure 3c).^[^ ^47^, ^48^ ^]^ Moreover, the absence of B_2g_ phonon modes, which result from translational symmetry breaking, proves that the spalled MoTe_2_ is a monolayer.^[^ ^49^ ^]^ It should be noted that no Ge‐related peak was observed, thus indicating that the water‐soluble Ge film was completely removed and that the spalled monolayer preserved its clean surface without any chemical contaminants (Figure S10 and S11, Supporting Information).^[^ ^50^, ^51^ ^]^ The Raman maps performed on 50 μm × 50 μm areas highlight the uniformity of the intensity of the A_1g_, E_2g_, and B_2g_ peaks (Figure 3d–f). Moreover, a strong PL peak was observed at 1.08 eV, as shown in Figure 3g.^[^ ^49^ ^]^

### 2.4 Electrical Properties in Spalled Monolayer MoTe_2_

**Figure 4:**
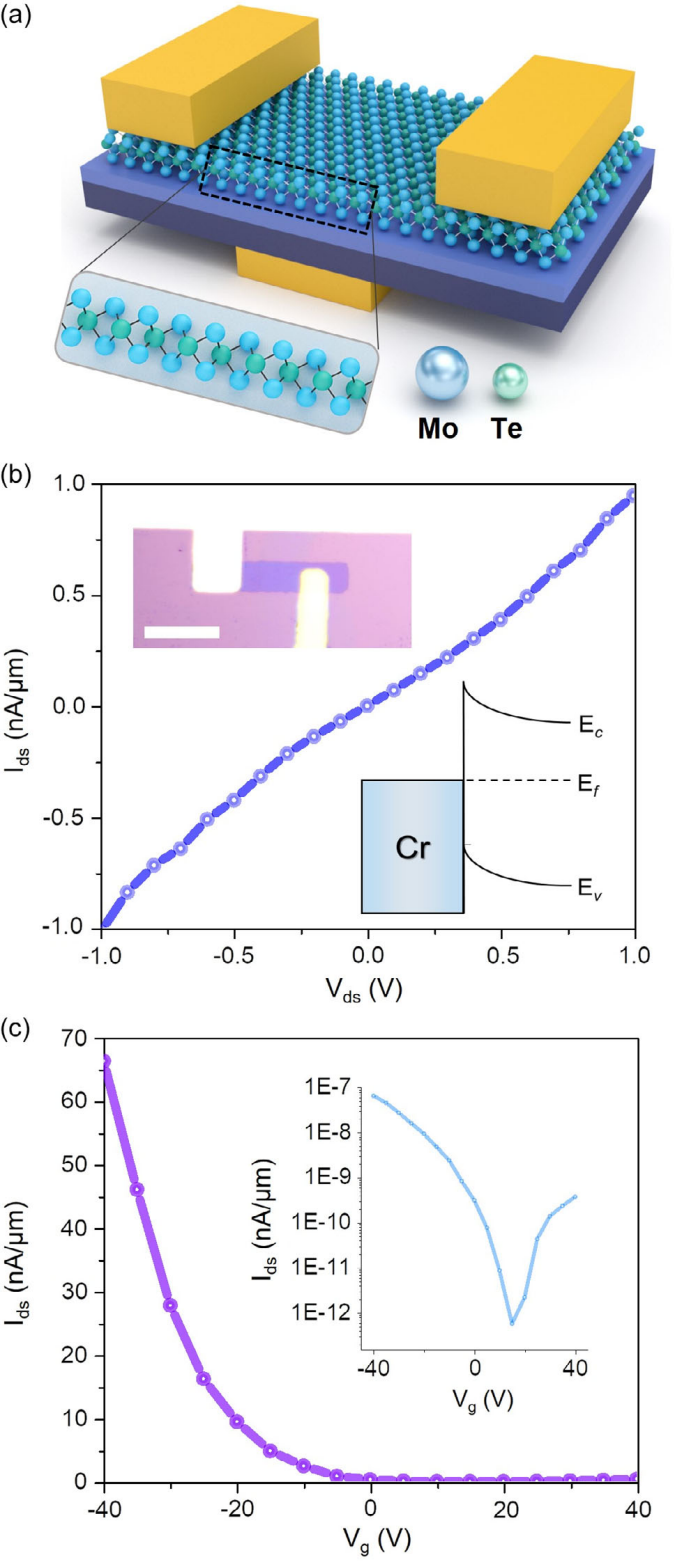
a) Schematic illustration of back‐gate monolayer MoTe_2_ FET. b) Output characteristics of spalled monolayer MoTe_2_ FET. An optical image of the device is shown in the inset. The scale bar is 10 μm. c) Transfer characteristics of spalled monolayer MoTe_2_ FET (*V*_ds_ = 0.5 V). The inset shows the logarithmic scale plot of the transfer curve.

Electrical measurements are one of the most effective methods for assessing the quality of layers. Therefore, by connecting the gate to the silicon, the field‐effect transistor (FET) properties of monolayer MoTe_2_ and MoS_2_ were investigated (Figure S12, Supporting Information). **Figure** **4**a demonstrates a schematic of back‐gated monolayer MoTe_2_ FETs, which is fabricated with an Au/Cr source and drain contacts on 300 nm SiO_2_/Si substrates. The detailed device fabrication process is described in the Experimental Section. Figure 4b shows that the results of source–drain current (*I*_ds_) vs source–drain voltage (*V*_ds_) (*I*_ds_–*V*_ds_) indicate the existence of Schottky contact between monolayer MoTe_2_ and metal electrodes, which is consistent with previous reports.^[^ ^52^ ^]^ The band alignment of the MoTe_2_ Schottky junction with a metal contact is illustrated in the inset of Figure 4b, providing an explanation for the Schottky contact in the *I*_ds_–*V*_ds_ results. Furthermore, Figure 4c shows the transfer characteristics of the monolayer MoTe_2_ FET device. The logarithmic plot of gate voltage (*V*_g_) in the inset in Figure 4c reveals the ambipolar behavior of monolayer MoTe_2_ devices.^[^ ^53^, ^54^ ^]^ These findings collectively suggest that the spalling process did not introduce chemical attack and contamination into the spalled monolayer MoTe_2_. By referring to the transfer plot, it is possible to calculate the field‐effect carrier mobility of a MoTe_2_ flake using $\mu_{\text{FE}}=\;\frac{L}{W}\frac{dI_{\text{ds}}}{C_{\text{ox}}V_{\text{ds}}dV_{g}}$, where *L* and *W* are the length and width of the channel (here, we used *L* = 7.5 μm and *W* = 4 μm), and *C*_ox_ is the gate oxide capacitance of 300 nm SiO_2_. The calculated mobility of MoTe_2_ is 30.4 cm^2^ V^−1^ s^−1^, which is comparable to the results of previous studies using mechanically exfoliated MoTe_2_ flakes (0.1–35.3 cm^2 ^V^−1 ^s^−1^), thus indicating that the spalling method is capable of preserving the quality of layers and is particularly advantageous for unstable materials, unlike other metal‐based modified exfoliation methods (Figure S9, Supporting Information).^[^ ^55^, ^56^, ^57^, ^58^ ^]^

## 3 Conclusions

This study presents a water‐soluble nonmetal Ge‐mediated atomic spalling approach that is a simple and facile method for obtaining large‐area monolayer TMDCs. A modified analytic model was used to calculate the effectiveness of the Ge stressor for selectively separating monolayer TMDC from bulk crystal. MoS_2_ and MoTe_2_ monolayers with lateral sizes of several millimeters were successfully achieved without needing to use an acid‐based wet‐etching process. Moreover, the study obtained a monolayer of graphene and other TMDCs (MoSe_2_ and WSe_2_) by applying the Ge‐mediated atomic spalling approach (Figure S13, Supporting Information). Detailed spectroscopic and electrical characterizations were conducted, that supported the high crystallinity of spalled monolayer TMDCs. These results have the potential to be widely employed for the high‐throughput manufacture of high‐quality monolayer TMDCs.

## 4 Experimental Section

4.1

4.1.1

##### Ge‐Mediated Spalling Process

Tightly fixed TMDC crystals (MoS_2_ and MoTe_2_, 2D semiconductors) with epoxy resin (Officeahn) were prepared. Next, 70 nm Ge film was deposited on the fresh surface of TMDC using e‐beam deposition. The deposition rate was set to 0.1 Å s^−1^ for the initial 5 nm of Ge to minimize physical damage and then increased to 1.1 Å s^−1^ for the remaining 65 nm of Ge film. Subsequently, poly(methyl methacrylate) (PMMA) was spin coated at 2000 rpm for 1 min. Thermal release tape (Revalpha 3196, Nitto Denko, TRT) was attached to the top of the PMMA/Ge/TMDC, and gentle force was applied to exfoliate the monolayer TMDC from the bulk TMDC and transfer it to the target substrate. The TRT lost its adhesive force at 110 °C and was removed from the substrate. The PMMA was removed by immersing the sample in acetone, after which the Ge film was removed in a deionized water bath at 60 °C.

##### Characterization

The surface morphology‐spalled samples were analyzed using bright‐field optical microscopy (BF‐OM, Olympus) and AFM (MFP‐3D Origin, Oxford Instruments). For the average roughness analysis of the surface (*R*_q_), tapping mode was performed at a slow scanning speed of 0.1 Hz, and the acquired AFM images were analyzed using Gwyddion software. Raman/PL spectroscopy (Renishaw Raman system) with excitation laser lines of 532 nm was used to characterize the crystallinity and quality of spalled monolayer. To prevent the heating effect of spalled monolayer, the power of the excitation laser line was kept below 0.1 mW. The intrinsic peak of Si, 520 cm^−1^, was used as a reference for the wave number calibration. The internal stress of Ge film (*σ*_Ge_) was calculated using the Stoney formula, $\sigma_{\text{Ge}}=\;\frac{1}{6}\left(\frac{1}{R}-\frac{1}{R_{0}}\right)\frac{Y_{s}t_{s}^{2}}{\left(1-\nu_{s}\right)t}$, where *R* is the measured curvature of Si after Ge film deposition, *R*_0_ is the measured curvature of the Si before Ge film deposition, *Y*_s_ is Young's modulus of the Si wafer, *ν*_s_ is Poisson's ratio of Si wafer, *t*_s_ is the thickness of Si wafer, and *t* is thickness of Ge film. We used a standard H‐terminated Si wafer to measure the internal stress of Ge film.

##### Device Fabrication and Characterization

MoTe_2_ FET was fabricated using a typical photolithography process. First, the metal electrode was patterned on transferred MoTe_2_, followed by the deposition of Cr/Au 5/45 nm using an e‐beam evaporator. After the lift‐off process, the channel was defined by reactive ion etching (SF_6_/Ar, 40/40 sccm, 100 W, 60 s). The electrical characteristics of the devices were investigated using a Keithley SCS‐4200 system and a probe station (MSTECH, MST5000) under ambient conditions.

## Conflict of Interest

The authors declare no conflict of interest.

## Supporting information

Supplementary Material

## Data Availability

The data that support the findings of this study are available from the corresponding author upon reasonable request.

## References

[1] D. Akinwande , C. Huyghebaert , C.-H. Wang , M. I. Serna , S. Goossens , L.-J. Li , H.-S. P. Wong , F. H. L. Koppens , Nature 2019, 573, 507. 31554977 10.1038/s41586-019-1573-9

[2] J. Wu , H. Ma , P. Yin , Y. Ge , Y. Zhang , L. Li , H. Zhang , H. Lin , Small Sci. 2021, 1, 2000053. 10.1002/smsc.202000053 PMC11935949 40213355

[3] N. Peimyoo , T. Deilmann , F. Withers , J. Escolar , D. Nutting , T. Taniguchi , K. Watanabe , A. Taghizadeh , M. F. Craciun , K. S. Thygesen , Nat. Nanotechnol. 2021, 16, 888. 34083771 10.1038/s41565-021-00916-1

[4] K. Cao , S. Feng , Y. Han , L. Gao , T. H. Ly , Z. Xu , Y. Lu , Nat. Commun. 2020, 11, 284. 31941941 10.1038/s41467-019-14130-0 PMC6962388

[5] N. Ubrig , E. Ponomarev , J. Zultak , D. Domaretskiy , V. Zólyomi , D. Terry , J. Howarth , I. Gutiérrez-Lezama , A. Zhukov , Z. R. Kudrynskyi , Z. D. Kovalyuk , A. Patané , T. Taniguchi , K. Watanabe , R. V. Gorbachev , V. I. Fal'ko , A. F. Morpurgo , Nat. Mater. 2020, 19, 299. 32015532 10.1038/s41563-019-0601-3

[6] J. Xu , L. Chen , Y.-W. Dai , Q. Cao , Q.-Q. Sun , S.-J. Ding , H. Zhu , D. W. Zhang , Sci. Adv. 2017, 3, 1602246. 10.1126/sciadv.1602246 PMC5438220 28560330

[7] T. Ahmed , J. Zha , K. K. Lin , H. C. Kuo , C. Tan , D. H. Lien , Adv. Mater. 2023, 35, 2208054. 10.1002/adma.202208054 36808659

[8] J. Zha , M. Luo , M. Ye , T. Ahmed , X. Yu , D. H. Lien , Q. He , D. Lei , J. C. Ho , J. Bullock , K. B. Crozier , C. Tan , Adv. Funct. Mater. 2022, 32, 2111970.

[9] Y. Shen , Z. Dong , Y. Sun , H. Guo , F. Wu , X. Li , J. Tang , J. Liu , X. Wu , H. Tian , T.-L. Ren , Adv. Mater. 2022, 34, 2201916. 10.1002/adma.202201916 35535757

[10] M. R. Kumar , S. Singh , H. M. Fahmy , N. K. Jaiswal , S. Akin , A. E. Shalan , S. Lanceros-Mendez , M. Salado , J. Power Sources 2023, 556, 232256.

[11] X. Lu , M. I. B. Utama , J. Lin , X. Gong , J. Zhang , Y. Zhao , S. T. Pantelides , J. Wang , Z. Dong , Z. Liu , W. Zhou , Q. Xiong , Nano Lett. 2014, 14, 2419. 24678857 10.1021/nl5000906

[12] J. Li , S. Wang , L. Li , Z. Wei , Q. Wang , H. Sun , J. Tian , Y. Guo , J. Liu , H. Yu , N. Li , G. Long , X. Bai , W. Yang , R. Yang , D. Shi , G. Zhang , Small Sci. 2022, 2, 2200062. 10.1002/smsc.202200062 PMC11935850 40213011

[13] M. Kim , J. Seo , J. Kim , J. S. Moon , J. Lee , J.-H. Kim , J. Kang , H. Park , ACS Nano 2021, 15, 3038. 33512141 10.1021/acsnano.0c09430

[14] A. Zafar , H. Nan , Z. Zafar , Z. Wu , J. Jiang , Y. You , Z. Ni , Nano Res. 2017, 10, 1608.

[15] W. Zhou , X. Zou , S. Najmaei , Z. Liu , Y. Shi , J. Kong , J. Lou , P. M. Ajayan , B. I. Yakobson , J.-C. Idrobo , Nano Lett. 2013, 13, 2615. 23659662 10.1021/nl4007479

[16] A. K. Geim , I. V. Grigorieva , Nature 2013, 499, 419. 23887427 10.1038/nature12385

[17] K. S. Novoselov , A. Mishchenko , A. Carvalho , A. H. C. Neto , Science 2016, 353, aac9439. 27471306 10.1126/science.aac9439

[18] Y. Huang , E. Sutter , N. N. Shi , J. Zheng , T. Yang , D. Englund , H.-J. Gao , P. Sutter , ACS Nano 2015, 9, 10612. 26336975 10.1021/acsnano.5b04258

[19] X. Zhang , Y. Li , W. Mu , W. Bai , X. Sun , M. Zhao , Z. Zhang , F. Shan , Z. Yang , 2D Mater. 2021, 8, 032002.

[20] G. Z. Magda , J. Pető , G. Dobrik , C. Hwang , L. P. Biró , L. Tapasztó , Sci. Rep. 2015, 5, 14714. 26443185 10.1038/srep14714 PMC4595767

[21] Y. Huang , Y.-H. Pan , R. Yang , L.-H. Bao , L. Meng , H.-L. Luo , Y.-Q. Cai , G.-D. Liu , W.-J. Zhao , Z. Zhou , L.-M. Wu , Z. L. Zhu , M. Huang , L.-W. Liu , L. Liu , P. Cheng , K.-H. Wu , S.-B. Tian , C.-Z. Gu , Y.-G. Shi , Y.-F. Guo , Z. G. Cheng , J.-P. Hu , L. Zhao , G.-H. Yang , E. Sutter , P. Sutter , Y.-L. Wang , W. Ji , X.-J. Zhou , et al., Nat. Commun. 2020, 11, 2453. 32415180 10.1038/s41467-020-16266-w PMC7228924

[22] S. B. Desai , S. R. Madhvapathy , M. Amani , D. Kiriya , M. Hettick , M. Tosun , Y. Zhou , M. Dubey , J. W. Ager III , D. Chrzan , A. Javey , Adv. Mater. 2016, 28, 4053. 27007751 10.1002/adma.201506171

[23] J. Shim , S.-H. Bae , W. Kong , D. Lee , K. Qiao , D. Nezich , Y. J. Park , R. Zhao , S. Sundaram , X. Li , H. Yeon , C. Choi , H. Kum , R. Yue , G. Zhou , Y. Ou , K. Lee , J. Moodera , X. Zhao , J.-H. Ahn , C. Hinkle , A. Ougazzaden , J. Kim , Science 2018, 362, 665. 30309906 10.1126/science.aat8126

[24] M. Velicky , G. E. Donnelly , W. R. Hendren , S. McFarland , D. Scullion , W. J. I. DeBenedetti , G. C. Correa , Y. Han , A. J. Wain , M. A. Hines , D. A. Muller , K. S. Novoselov , H. D. Abruna , R. M. Bowman , E. J. G. Santos , F. Huang , ACS Nano 2018, 12, 10463. 30265515 10.1021/acsnano.8b06101

[25] A. C. Johnston , S. I. Khondaker , Adv. Mater. Interfaces 2022, 9, 2200106.

[26] F. Liu , W. Wu , Y. Bai , S. H. Chae , Q. Li , J. Wang , J. Hone , X.-Y. Zhu , Science 2020, 367, 903. 32079769 10.1126/science.aba1416

[27] M. Heyl , E. J. W. List-Kratochvil , Appl. Phys. A 2023, 129, 16. 10.1007/s00339-023-06492-6 PMC9977711 36876320

[28] J.-Y. Moon , D.-H. Kim , S.-I. Kim , H.-S. Hwang , J.-H. Choi , S.-K. Hyeong , S. Ghods , H. G. Park , E.-T. Kim , S. Bae , S.-K. Lee , S.-K. Son , J.-H. Lee , Matter 2022, 5, 3935.

[29] J.-Y. Moon , M. Kim , S.-I. Kim , S. Xu , J.-H. Choi , D. Whang , K. Watanabe , T. Taniguchi , D. S. Park , J. Seo , S. H. Cho , S.-K. Son , J.-H. Lee , Sci. Adv. 2020, 6, eabc6601. 33115746 10.1126/sciadv.abc6601 PMC7608796

[30] X. Wang , Y. Sun , K. Liu , 2D Mater. 2019, 6, 042001.

[31] C. Tayran , S. Aydin , M. Cakmak , Ş. Ellialtıoğlu , Solid State Commun. 2016, 231, 57.

[32] M. C. Schabel , J. L. Martins , Phys. Rev. B 1992, 46, 7185. 10.1103/physrevb.46.7185 10002428

[33] J. W. Hutchinson , Z. Suo , Adv. Appl. Mech. 1991, 29, 63.

[34] H. Park , C. Lim , C.-J. Lee , M. Choi , S. Jung , H. Park , Solid-State Electron. 2020, 163, 107660.

[35] H. Li , Q. Zhang , C. C. R. Yap , B. K. Tay , T. H. T. Edwin , A. Olivier , D. Baillargeat , Adv. Funct. Mater. 2012, 22, 1385.

[36] Y. Zhou , J. Zhang , E. Song , J. Lin , J. Zhou , K. Suenaga , W. Zhou , Z. Liu , J. Liu , J. Lou , H. J. Fan , Nat. Commun. 2020, 11, 2253. 32382108 10.1038/s41467-020-16111-0 PMC7205865

[37] S. Mignuzzi , A. J. Pollard , N. Bonini , B. Brennan , I. S. Gilmore , M. A. Pimenta , D. Richards , D. Roy , Phys. Rev. B 2015, 91, 195411.

[38] S. Tongay , J. Suh , C. Ataca , W. Fan , A. Luce , J. S. Kang , J. Liu , C. Ko , R. Raghunathanan , J. Zhou , F. Ogletree , J. Li , J. C. Grossman , J. Wu , Sci. Rep. 2013, 3, 2657. 24029823 10.1038/srep02657 PMC3772378

[39] A. Splendiani , L. Sun , Y. Zhang , T. Li , J. Kim , C.-Y. Chim , G. Galli , F. Wang , Nano Lett. 2010, 10, 1271. 20229981 10.1021/nl903868w

[40] Y. Kim , J.-G. Song , Y. J. Park , G. H. Ryu , S. J. Lee , J. S. Kim , P. J. Jeon , C. W. Lee , W. J. Woo , T. Choi , H. Jung , H.-B.-R. Lee , J.-M. Myoung , S. Im , Z. Lee , J.-H. Ahn , J. Park , H. Kim , Sci. Rep. 2016, 6, 18754. 10.1038/srep18754 PMC4698672 26725854

[41] Z. Yin , H. Li , H. Li , L. Jiang , Y. Shi , Y. Sun , G. Lu , Q. Zhang , X. Chen , H. Zhang , ACS Nano 2012, 6, 74. 22165908 10.1021/nn2024557

[42] B. Chen , H. Sahin , A. Suslu , L. Ding , M. I. Bertoni , F. M. Peeters , S. Tongay , ACS Nano 2015, 9, 5326. 25868985 10.1021/acsnano.5b00985

[43] G. Cunningham , M. Lotya , C. S. Cucinotta , S. Sanvito , S. D. Bergin , R. Menzel , M. S. P. Shaffer , J. N. Coleman , ACS Nano 2012, 6, 3468. 22394330 10.1021/nn300503e

[44] S. Song , D. H. Keum , S. Cho , D. Perello , Y. Kim , Y. H. Lee , Nano Lett. 2016, 16, 188. 26713902 10.1021/acs.nanolett.5b03481

[45] L. Sun , L. Lin , Z. Wang , D. Rui , Z. Yu , J. Zhang , Y. Li , X. Liu , K. Jia , K. Wang , L. Zheng , B. Deng , T. Ma , N. Kang , H. Xu , K. S. Novoselov , H. Peng , Z. Liu , Adv. Mater. 2019, 31, 1902978. 10.1002/adma.201902978 31502709

[46] G. Mirabelli , C. McGeough , M. Schmidt , E. K. McCarthy , S. Monaghan , I. M. Povey , M. McCarthy , F. Gity , R. Nagle , G. Hughes , A. Cafolla , P. K. Hurley , J. Appl. Phys. 2016, 120, 125102.

[47] J.-H. Li , D. Bing , Z.-T. Wu , G.-Q. Wu , J. Bai , R.-X. Du , Z.-Q. Qi , Chin. Phys. B 2020, 29, 017802.

[48] G. Froehlicher , E. Lorchat , F. Fernique , C. Joshi , A. Molina-Sánchez , L. Wirtz , S. Berciaud , Nano Lett. 2015, 15, 6481. 26371970 10.1021/acs.nanolett.5b02683

[49] C. Ruppert , B. Aslan , T. F. Heinz , Nano Lett. 2014, 14, 6231. 25302768 10.1021/nl502557g

[50] S. Kanakaraju , A. K. Sood , S. Mohan , J. Appl. Phys. 1998, 84, 5756.

[51] T. Tah , C. K. Singh , S. Amirthapandian , K. K. Madapu , A. Sagdeo , S. Ilango , T. Mathews , S. Dash , Mater. Sci. Semicond. Process. 2018, 80, 31.

[52] T. Kim , D. Joung , J. Park , Curr. Appl. Phys. 2018, 18, 843.

[53] S. Fathipour , N. Ma , W. S. Hwang , V. Protasenko , S. Vishwanath , H. G. Xing , H. Xu , D. Jena , J. Appenzeller , A. Seabaugh , Appl. Phys. Lett. 2014, 105, 192101.

[54] Y.-F. Lin , Y. Xu , S.-T. Wang , S.-L. Li , M. Yamamoto , A. Aparecido-Ferreira , W. Li , H. Sun , S. Nakaharai , W.-B. Jian , K. Ueno , K. Tsukagoshi , Adv. Mater. 2014, 26, 3263. 24692079 10.1002/adma.201305845

[55] P. H. Nguyen , D. H. Nguyen , H. Kim , H. M. Jeong , H. M. Oh , M. S. Jeong , Appl. Surf. Sci. 2022, 596, 153567.

[56] V. K. Nagareddy , T. J. Octon , N. J. Townsend , S. Russo , M. F. Craciun , C. D. Wright , Adv. Funct. Mater. 2018, 28, 1804434.

[57] S. Larentis , B. Fallahazad , H. C. P. Movva , K. Kim , A. Rai , T. Taniguchi , K. Watanabe , S. K. Banerjee , E. Tutuc , ACS Nano 2017, 11, 4832. 28414214 10.1021/acsnano.7b01306

[58] Q. He , P. Li , Z. Wu , B. Yuan , Z. Luo , W. Yang , J. Liu , G. Cao , W. Zhang , Y. Shen , P. Zhang , S. Liu , G. Shao , Z. Yao , Adv. Mater. 2019, 31, 1901578. 10.1002/adma.201901578 31199026

