# Supplementary material for: Nonmetal‐Mediated Atomic Spalling of Large‐Area Monolayer Transition Metal Dichalcogenide
Source: Small Sci. 2023 Jun 30;3(9):2300033. doi: 10.1002/smsc.202300033 (PMC11935789; doi:10.1002/smsc.202300033)
Supplement: Supplementary file 1 — Supplementary Material [file SMSC-3-2300033-s001.pdf]

Supporting Information

Nonmetal-Mediated Atomic Spalling of Large-Area Monolayer Transition-Metal Dichalcogenide

Sein Kim[1,#], Seung-Il Kim[1,#], Soheil Ghods[1], Jin-Su Kim[2], Young Cheol Lee[2], Hyung Jun Kwun[2], Ji-Yun Moon[1,] and Jae-Hyun Lee[1,2,]**

S. Kim, S.-I. Kim, Dr. S. Ghods, J.-Y. Moon, and Prof. J.-H. Lee
[1] Department of Energy Systems Research, Ajou University, Suwon 16499, Republic of Korea

J.-S. Kim, Y. C. Lee, H. J. Kwun, and Prof. J.-H. Lee
[2] Department of Materials Science and Engineering, Ajou University, Suwon 16499, Republic of Korea

[*] Corresponding Authors: ydnas96@ajou.ac.kr (J.-Y. Moon) and jaehyunlee@ajou.ac.kr (J.-H. Lee)

Note S1: Critical spalling conditions of Ge/TMDCs bilayer system

The surface-layer spalling model of the modified Suo-Hutchinson (S-H) model considers sub-surface cracks of a semi-infinite plate.[1] We calculated the critical spalling conditions in a bilayer Ge/TMDCs system. According to the surface layer spalling model, the stress intensity factors satisfying the plane strain condition are expressed as follows:

$$K_{\mathrm{I}} = \frac{\sigma_{\mathrm{f}} t_{\mathrm{f}}}{\sqrt{2\left(t_{\mathrm{f}}+d_{\mathrm{spall}}\right)}}\left[cos(\omega) + \sqrt{3}\left(1-\frac{t_{\mathrm{f}}}{t_{\mathrm{f}}+d_{\mathrm{spall}}}\right) sin(\omega)\right] \tag{1}$$

$$K_{\mathrm{II}} = \frac{\sigma_{\mathrm{f}} t_{\mathrm{f}}}{\sqrt{2\left(t_{\mathrm{f}}+d_{\mathrm{spall}}\right)}}\left[sin(\omega) - \sqrt{3}\left(1-\frac{t_{\mathrm{f}}}{t_{\mathrm{f}}+d_{\mathrm{spall}}}\right) cos(\omega)\right] \tag{2}$$

In mixed mode fracture, the resulting equation for the two stress intensity factors, vertical stress direction (K_{I}) and lateral stress direction (K_{II}), is given where, t_{f}, σ_{f} and d_{spall} are the thickness of the Ge stressor film, the internal stress of the Ge stressor film, and crack propagation depth, respectively.[1] Since the plane strain condition is satisfied, ω = 52.07 °. When a specific external stress is applied to the Ge stressor film, a crack is initiated from the edge of the TMDCs crystal. Subsequently, the crack propagates downward due to the mixed-mode fracture of K_{I} and K_{II}. When $K_{\mathrm{II}} = 0$, cracks propagate parallel to the interface between the Ge stressor film and the TMDCs crystal. The spalling depth is expressed as follows.

$$d_{\mathrm{spall}} = \left[\frac{tan(\omega)}{\sqrt{3}-tan(\omega)}\right] t_{\mathrm{f}} = 2.86 t_{\mathrm{f}} \tag{3}$$

Since $K_{\mathrm{II}} = 0$, it is necessary that K_{I} be greater than or equal to the critical stress intensity of the substrate (K_{IC}). This equation can be expressed as follows:

$$K_{\mathrm{I}} \geq K_{\mathrm{IC}} \tag{4}$$

Using the Equation (1) to (4), the subcritical residual stress (σ_{c}) can be calculated as:

$$\sigma_{\mathrm{c}} = \frac{\left[K_{\mathrm{IC}}\left(2\sqrt{t_{\mathrm{f}}+d_{\mathrm{spall}}}\right)\right]}{t_{\mathrm{f}}\left[cos(\omega)+\sqrt{3}\left(1-\frac{t_{\mathrm{f}}}{t_{\mathrm{f}}+d_{\mathrm{spall}}}\right)sin(\omega)\right]} \tag{5}$$

When a material undergoes deformation or cracks due to external forces, the spalling of the Ge/TMDCs bilayer system can be described by fracture mechanics, satisfying Mode I: opening, and the critical energy release rate (G_{c}) is expressed as follows:

$$G_{\mathrm{C}} = \frac{{K_{\mathrm{IC}}}^{2}}{\bar{E}} \tag{6}$$

Where, $\overline{E}$ is the relative Young's modulus of TMDCs, can be represented by the following equation:

$$\overline{E} = \frac{Y_s}{1\text{-}{\nu_s}^2} \quad (7)$$

Where, Y_s is Young's modulus of TMDCs, ν_s is Poisson's ratio, respectively. Consequently, K_{IC} can be calculated as follows:[1, 2]

$$K_{\mathrm{IC}}=\sqrt{G_{\mathrm{C}}\frac{Y_s}{1\text{-}{\nu_s}^2}} \quad (8)$$

We calculated critical spalling conditions using the mechanical properties of MoS_2 crystal (here, we used ν_s = 0.22, G_c = 0.45 J/m^2 and Y_s = 2.669 GPa).[3-6]

Based on our previous study, which confirmed that the value of K_{IC} for MoS_2 is 35.5 KPam$^{1/2}$, we calculated the theoretical relationship between the internal stress and the thickness of the Ge stressor film. According to Equation (8), the lower boundary represents the subcritical boundary within the regime where spalling does not occur, while the upper boundary corresponds to the spontaneous boundary at which spontaneous spalling occurs. Accordingly, our spontaneous boundary calculations resulted in a value three times larger than Gc.[1, 7, 8] We measured the internal stress value (262 ± 10 Mpa) that satisfied the controlled spalling window at a fixed Ge stressor film thickness (~70 nm) and employed it as a monolayer spalling condition.

Note S2: Modifying crack-propagation depth for monolayer spalling

We theoretically calculated the dependence of the spalling depth according to the TMDCs substrate thickness. Our previous work demonstrated the correlation between the total accumulated strain energy and the crack-propagation depth.[6] The elastic strain energy accumulated in the Ge stressor film (U_f) and MoS2 crystal (U_s) can be calculated by the following equation, respectively:[9]

$$U_f = \frac{(1\text{-}v_f)}{2Y_f} t_f \sigma_f^2 \tag{9}$$

$$U_s = \frac{(1\text{-}v_s)}{2Y_s}\frac{t_f^2}{t_s}\sigma_f^2 \left[\frac{7}{2} \text{-}12\left(\frac{y_s}{t_s}\right)^3 \text{-}6\left(\frac{y_s}{t_s}\right)^2 \text{-} \left(\frac{y_s}{t_s}\right)\right] \tag{10}$$

Where, σ, v, t, and Y are the internal stress, Poisson's ratio, thickness, elastic strain and Young's modulus of the Ge stressor film (f) and MoS_2 substrate (s), respectively. $y_s = t_s/2\text{-}d_{spall}$, the point of spalling initiation. It is confirmed that the strain energy accumulated in MoS_2 crystal depends on the the crystal thickness as well as the crack propagation depth.[9] The total accumulated strain energy, U_{Total} can be defined as follows:

$$U_{Total} = U_f + U_s = \frac{(1\text{-}v_f)}{2Y_f} t_f \sigma_f^2 + \frac{(1\text{-}v_s)}{2Y_s}\frac{t_f^2}{t_s}\sigma_f^2 \left[12\left(\frac{d_{spall}}{t_s}\right)^3 \text{-}24\left(\frac{d_{spall}}{t_s}\right)^2 +16\left(\frac{d_{spall}}{t_s}\right)\right] \tag{11}$$

When the total accumulated strain energy reaches the weak bonding energy value in the MoS_2 crystal (γ), spalling occurs, so Equation (11) is converted as follows:

$$\sigma_f = \sqrt{\frac{\gamma}{\frac{(1\text{-}v_s)t_f^2}{2Y_s\ t_s}\left[12\left(\frac{d_{spall}}{t_s}\right)^3 \text{-}24\left(\frac{d_{spall}}{t_s}\right)^2 +16\left(\frac{d_{spall}}{t_s}\right)\right] + \frac{(1\text{-}v_f)}{2Y_f}t_f}} \tag{12}$$

(here, we used Y_f = 83 GPa, v_f = 0.39, t_f = 70 nm, and γ = 34.87 mJ/m^2).[10, 11] This approach confirms that the thermodynamic equilibrium condition of the total accumulated strain energy and the weak binding energy of the MoS_2 crystal is primarily determined by the thickness of the MoS_2 crystal at a fixed critical spalling condition. When the thickness of the MoS_2 crystal is between 170 nm and 230 nm, it offers a range of spalling thicknesses for monolayer MoS_2 (Figure 1b). Based on the theoretically calculated spalling depth, we verified the spalling of monolayers from MoS_2 crystals with an average thickness of 192 ± 27 nm. Direct experimental validation of computational models enables estimation of monolayer TMDCs (MoS_2 and $MoTe_2$) separations.

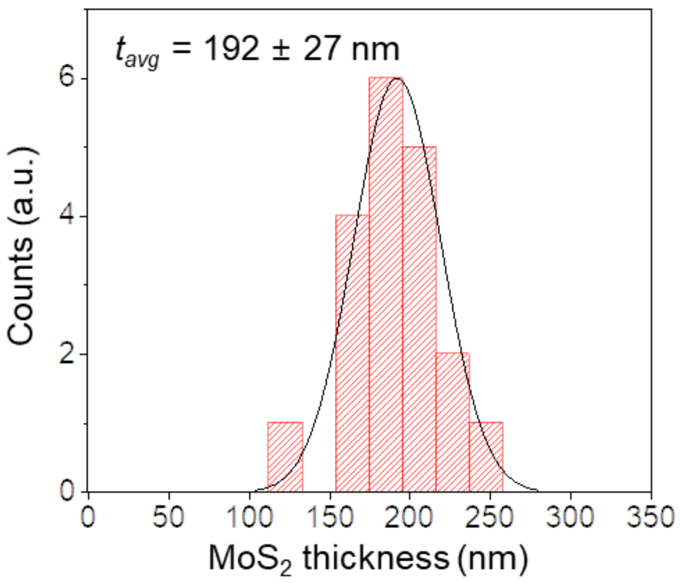

Figure S1. Histograms of the height of the cleaved MoS_2 crystal for 20 samples.

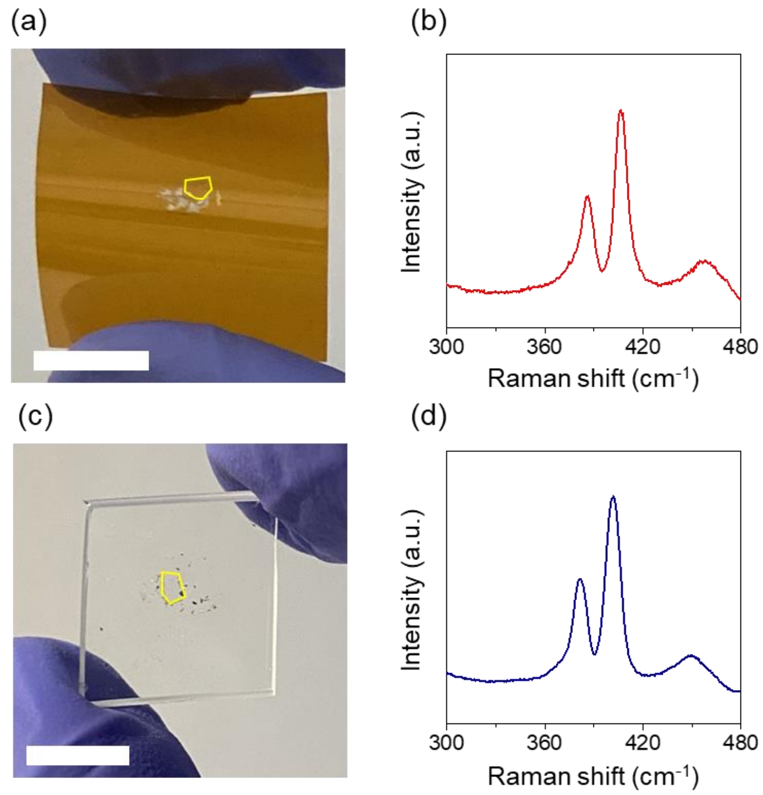

Figure S2. Photographs and Raman spectra of spalled MoS_2 on (a) PI substrate and (b) PDMS substrate. Scale bars are 1 cm and 0.5 cm, respectively.

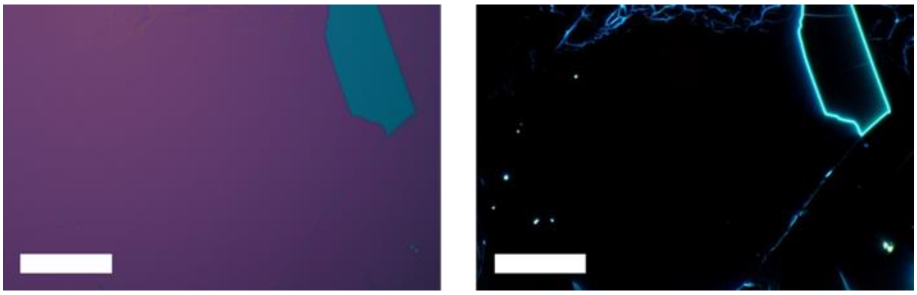

Figure S3. High magnification (Left) bright field and (Right) dark field OM images of spalled MoS_2 on 300 nm SiO_2/Si substrate. Scale bar is 25 μm.

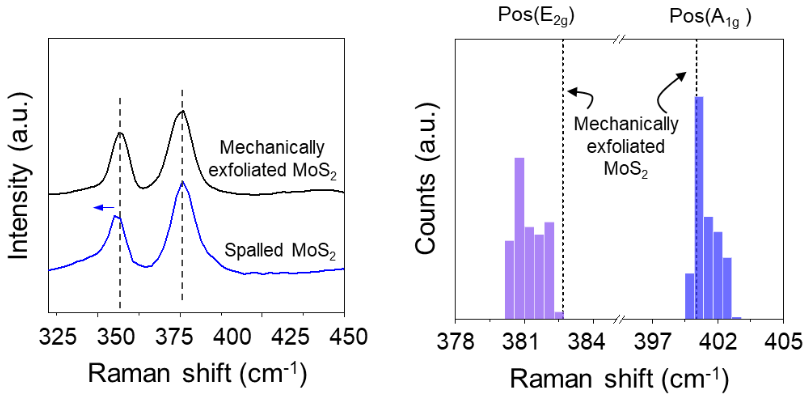

Figure S4. (Left) Representative Raman spectra of tape exfoliated- and spalled monolayer MoS_2**.** (Right) Histogram of peak positions (E_{2g} and A_{1g}) of spalled monolayer MoS_2. Black dotted line indicates the position of E_{2g} and A_{1g} peaks of tape exfoliated monolayer MoS_2.

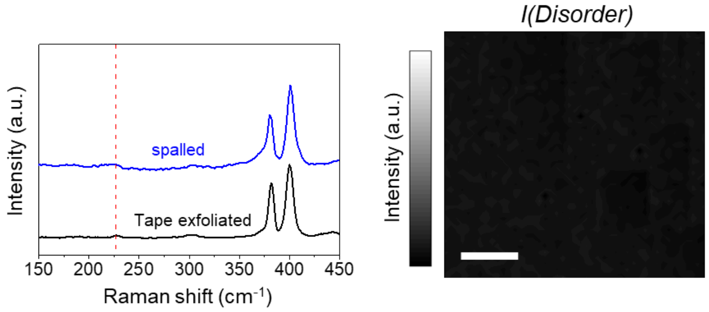

Figure S5. (left) Raman spectrum of spalled monolayer MoS_2 and tape exfoliated monolayer MoS_2 on 300 nm SiO_2/Si substrate. Red dashed line indicates the position of defect related Raman peak. (right) Raman maps of intensity of defect-related peak in spalled monolayer MoS_2 on 300 nm SiO_2/Si substrate. scale bar is 10 μm.

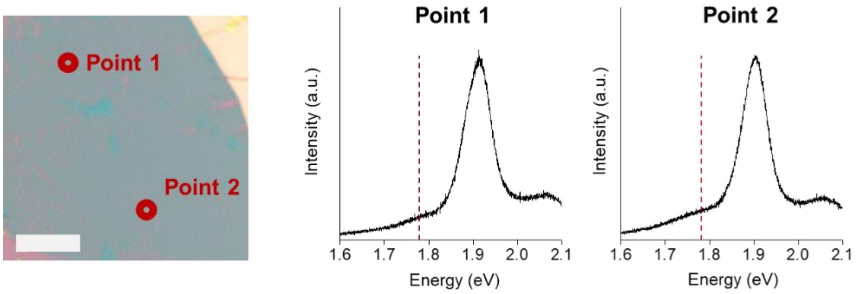

Figure S6. OM images of spalled monolayer MoS_2 monolayer on 300 nm SiO_2/Si substrate. Scale bar is 100 μm. Low temperature PL analysis conducted at different point marked in the OM images (red circle symbol) and defect-related PL peak at 1.78 eV was not observed.

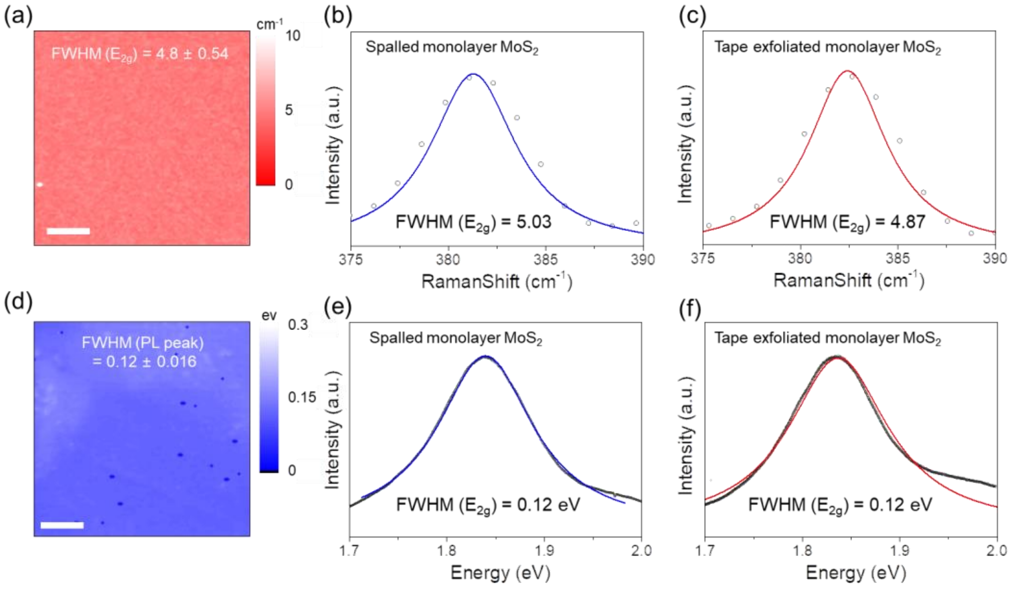

Figure S7. a) Raman maps of FWHM (E_{2g}) for spalled monolayer MoS_2 on 300 nm SiO_2/Si substrate. Scale bar is 10 µm. b, c) FWHM of E_{2g} peak for spalled-, and tape exfoliated monolayer MoS_2 on 300 nm SiO_2/Si substrates, respectively. d) PL peak FWHM maps for spalled monolayer MoS_2 on 300 nm SiO_2. Scale bar is 10 µm. e, f) FWHM of PL peak for spalled-, and tape exfoliated monolayer MoS_2 on 300 nm SiO_2/Si substrates, respectively.

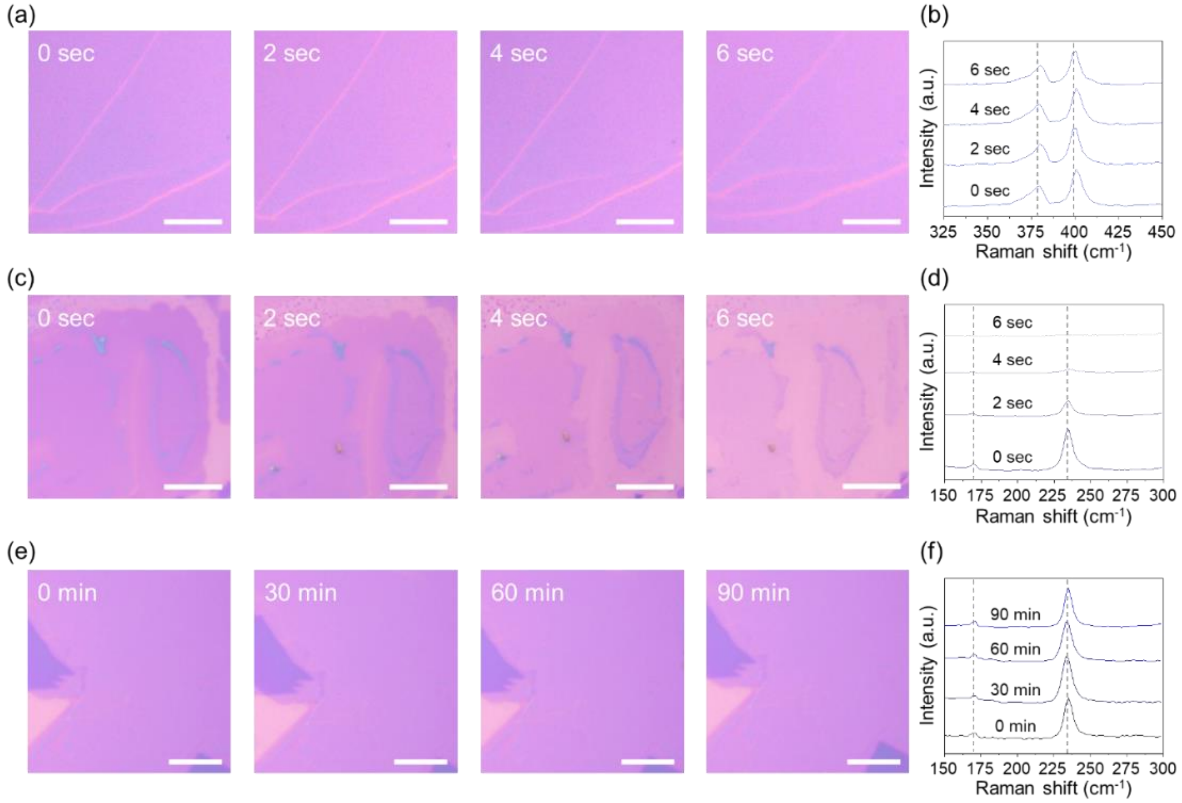

Figure S8. Time-dependent optical images and Raman spectra of (a) MoS_2 and (b) $MoTe_2$ dipped in the conventional metal etchant and (c) $MoTe_2$ dipped in the DI water. Scale bar is 10 μm. Spalled monolayer MoS_2 exhibits stability in the conventional metal etchant with no remarkable changes, whereas spalled monolayer $MoTe_2$ exhibits a rapid optical contrast and a gradually decreased intensity of Raman spectra within a few seconds. On the contrary, a pristine state of spalled monolayer $MoTe_2$ maintained ever over the dipping in DI water for 90 minutes.

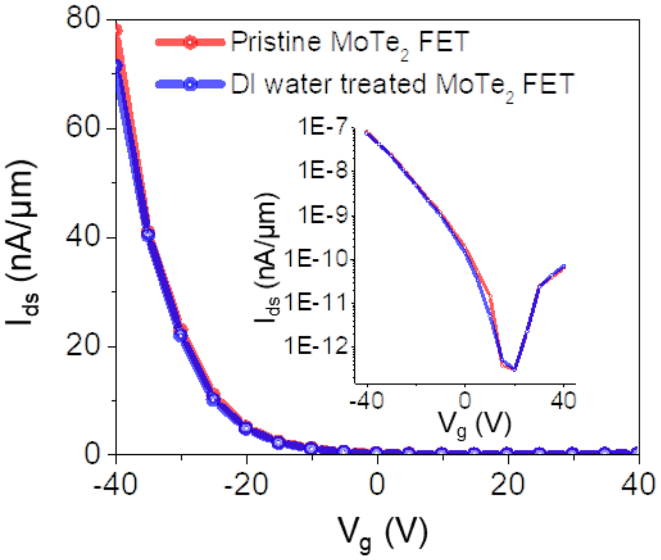

Figure S9. Transfer characteristics of tape exfoliated monolayer $MoTe_2$ FET before and after dipping in DI water for 90 min. The carrier mobility of $MoTe_2$ FET before/after dipping in DI water were calculated as 35.3 $cm^2V^{-1}s^{-1}$ and 32.1 $cm^2V^{-1}s^{-1}$, respectively.

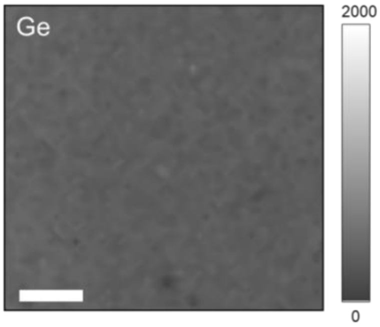

Figure S10. Raman map for Ge related peak at 292 cm^{-1} of spalled monolayer $MoTe_2$. Scale bar is 10 μm.

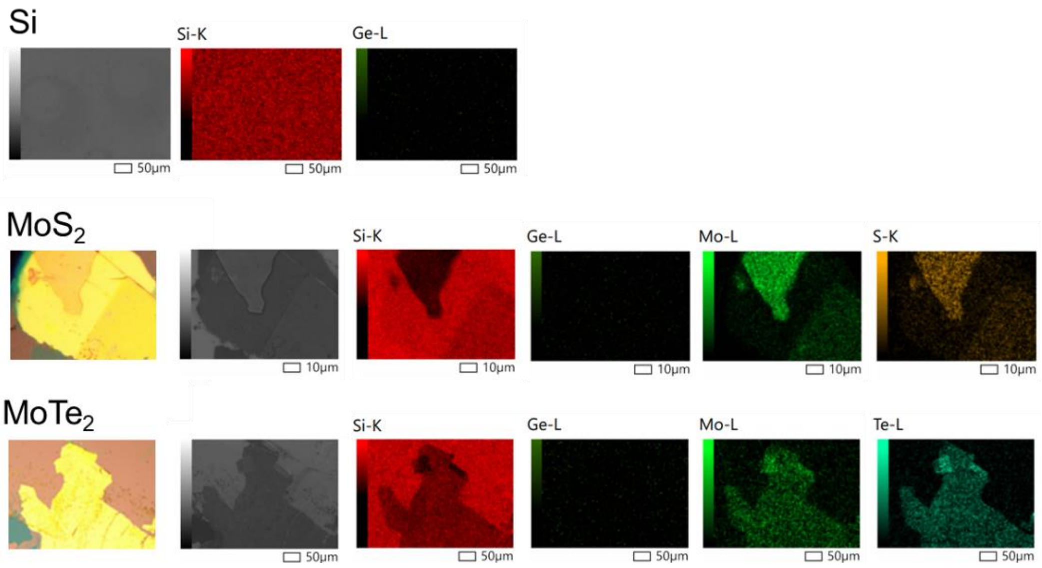

Figure S11. OM images and corresponding SEM and EDAX maps of bare Si, spalled MoS_2 and $MoTe_2$, respectively. EDAX chemical maps for spalled TMDCs show the spatial distribution of Si, Ge, Mo, S and Te elements, respectively.

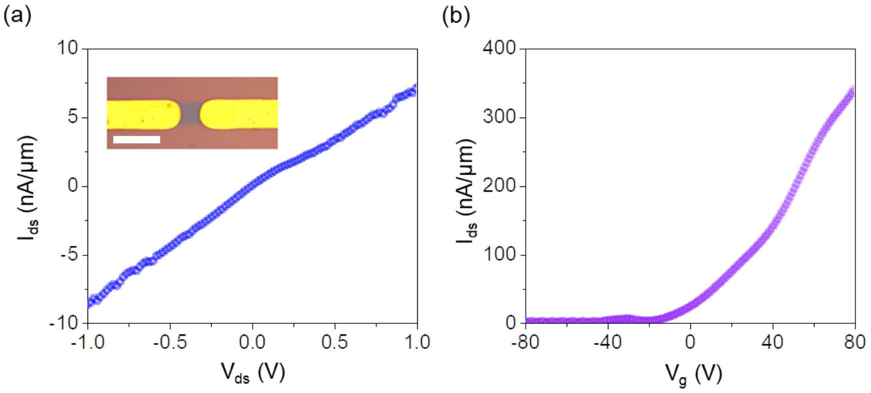

Figure S12. a) Output characteristic of spalled monolayer MoS_2 FET. Inset shows the optical image of the device. Scale bar is 10 µm and the channel area is defined as length = 7 µm and width = 5 µm. b) Transfer characteristics of spalled monolayer MoS_2 FET (V_{ds} = 2 V). The carrier mobility was calculated to be 22.5 $cm^2V^{-1}s^{-1}$

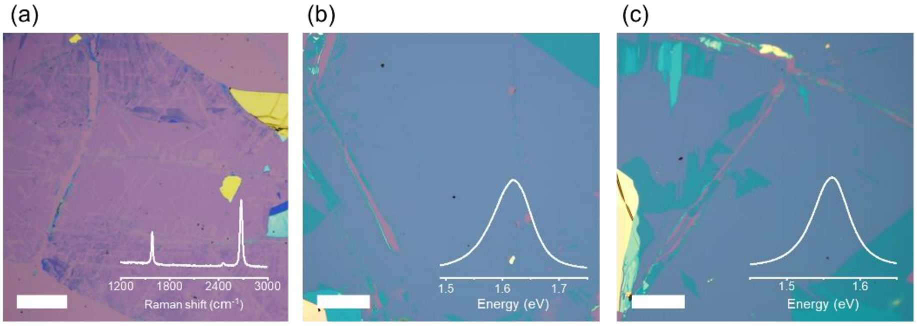

Figure S13. Optical images of large-scale spalled monolayer a) graphene b) $MoSe_2$ and c) WSe_2. Scale bar is 100 μm. Insets in a) to c) show Raman and PL spectra of each spalled samples. The high I_{2D}/I_G ratio of graphene and strong PL intensity of $MoSe_2$ and WSe_2 were observed, confirming the nature of the monolayer.

References

[1] J. W. Hutchinson, Z. Suo *Adv. Appl. Mech.* **1991**, *29*, 63.

[2] K.-J. Soderholm *Dent. Mater.* **2010**, *26*, e63.

[3] J. Shim, S.-H. Bae, W. Kong, D. Lee, K. Qiao, D. Nezich, Y. J. Park, R. Zhao, S. Sundaram, X. Li, H. Yeon, C. Choi, H. Kum, R. Yue, G. Zhou, Y. Ou, K. Lee, J. Moodera, X. Zhao, J.-H. Ahn, C. Hinkle, A. Ougazzaden, J. Kim, *Science* **2018**, *362*, 665.

[4] R. Gaillac, P. Pullumbi, F.-X. Coudert *J. Phys.: Condens. Matter* **2016**, *28*, 275201.

[5] B. Hajgato, S. Güryel, Y. Dauphin, J.-M. Blairon, H. E. Miltner, G. Van Lier, F. De Proft, P. Geerlings *Chem. Phys. Lett.* **2013**, *564*, 37.

[6] J.-Y. Moon, D.-H. Kim, S.-I. Kim, H.-S. Hwang, J.-H. Choi, S.-K. Hyeong, S. Ghods, H. G. Park, E.-T. Kim, S. Bae, S.-K. Lee, S.-K. Son, J.-H. Lee, *Matter* **2022**, *5*, 3935.

[7] A. G. Evans, M. D. Drory, M. S. Hu *J. Mater. Res.* **1988**, *3*, 1043.

[8] D. Crouse, J. Simon, K. L. Schulte, D. L. Young, A. J. Ptak, C. E. Packard *Thin Solid Films* **2018**, *649*, 154.

[9] H. Park, C. Lim, C.-J. Lee, M. Choi, S. Jung, H. Park *Solid-State Electron.* **2020**, *163*, 107660.

[10] E. B. T. Walch, C. Roos *Int. J. Appl. Glass Sci.* **2020**, *11*, 195.

[11] M. Annamalai, K. Gopinadhan, S. A. Han, S. Saha, H. J. Park, E. B. Cho, B. Kumar, A. Patra, S.-W. Kim, T. Venkatesan *Nanoscale* **2016**, *8*, 5764.
